# Capacidad de autocuidado de adultos con fibrosis quística en la transición/transferencia entre servicios

**DOI:** 10.23938/ASSN.1084

**Published:** 2024-09-15

**Authors:** Amalia Coca Barbado, Ester Zamarrón de Lucas, Rodolfo Álvarez-Sala Walther, María Concepción Prados Sánchez

**Affiliations:** 1 Universidad Autónoma de Madrid Facultad de Medicina y Cirugía Departamento de Medicina Madrid España; 2 Servicio Madrileño de Salud Hospital Universitario la Paz Servicio de Neumología Madrid España

**Keywords:** Fibrosis Quística, Autocuidado, Transición a la atención de adultos, Continuidad de la atención al paciente, Cystic fibrosis, Self-care, Transition to adult care, Continuity of patient care

## Abstract

**Fundamento:**

Analizar los conocimientos, habilidades y estado emocional de los pacientes con fibrosis quística (FQ) en un momento del seguimiento, comparándolos con el recuerdo que tenían de la transición (paso planificado y progresivo desde la unidad infantil) / transferencia (traspaso sin mediar los pasos recomendados por las guías) a una unidad especializada de FQ de adultos.

**Material y métodos:**

Estudio prospectivo transversal sobre adultos con FQ en seguimiento en una consulta especializada. El grupo 1 realizó la transición y el grupo 2 fue transferido directamente. Se recogieron variables sociodemográficas, grado de conocimientos y habilidades, y estado emocional mediante una encuesta diseñada *ad hoc* (la validación de la consistencia interna fue parte del estudio) y la subescala emocional del Cuestionario Revisado de Calidad de Vida para FQ. Se compararon ambos grupos en la transición/transferencia y en el seguimiento, así como la evolución entre ambos momentos.

**Resultados:**

Se incluyeron 35 personas con FQ, 65,8% hombres y edad media 31,9 años (DE=10,1). En la transición, el grupo 1 (n=19; 54,3%) mostró mayores conocimientos sobre su medicación, aunque menores habilidades para gestionar citas y tomar decisiones que el grupo 2 en la transferencia. En la visita de seguimiento, el grupo 1 describió mejor estado emocional. Durante el seguimiento, el grupo 1 mejoró significativamente sus habilidades de gestión de citas, comunicación y toma de decisiones.

**Conclusiones:**

Los pacientes que transicionaron a una unidad de adultos de FQ conocían mejor sus medicamentos, mientras que los transferidos gestionaban mejor sus citas y la toma de decisiones, pero se sentían más tristes.

## Abstract (en)

**Background:**

To analyze the knowledge, abilities, and emotional state of cystic fibrosis patients during a specific follow-up period and compare this with the recall they had of the transition (planned and gradual shift from the pediatric unit) / transfer (direct change skipping the steps recommended by the guidelines) to a specialized cystic fibrosis adult unit.

**Methods:**

Prospective cross-sectional study with cystic fibrosis adult patients under follow-up in a specialist consultation. Group 1 were patients who transitioned and Group 2 were transferred patients. The following information was collected: sociodemographic variables, degree of knowledge, skills, and emotional state using a survey designed for this purpose (as part of the internal consistency validation process). Participants also completed the emotional subscale of Cystic Fibrosis Questionnaire-Revised. Inter-group comparisons were made for the transition/transfer, at the follow-up, and during the evolution.

**Results:**

Thirty-five patients were analyzed; 65.8% male; mean age 31.9 years (SD =10.1). At the transition, Group 1 (n=19; 54.3%) had greater knowledge about their medication and reduced ability to manage appointments and making decisions in comparison to Group 2 at transfer. At follow-up, Group 1 made a better report on their emotional state and significantly improved their ability to manage appointments, communication, and decision-making.

**Conclusions:**

Patients who were moved to an adult cystic fibrosis unit through transition were more knowledgeable about their medications. However, those who were transferred managed their appointments and decision-making better, but felt sadder.

## INTRODUCCIÓN

La fibrosis quística (FQ) es la enfermedad hereditaria autosómica recesiva multisistémica más frecuente en la raza blanca. El grado de afectación pulmonar influye notablemente en el pronóstico de la enfermedad, puesto que es la causa principal de morbilidad y mortalidad^1^. En las últimas seis décadas, la instauración de programas de cribado neonatal, el impulso de unidades especializadas en FQ, los avances en trasplantes y el desarrollo de nuevos medicamentos, han supuesto un aumento considerable de la esperanza de vida^2^^,^^3^.

La persona joven con FQ debe afrontar, además de los cambios físicos y psicológicos inherentes a su etapa vital, los asociados a la transición de la atención pediátrica a la de adultos^4^^,^^5^. La Sociedad de Medicina del Adolescente^6^ define esta transición como un proceso activo que atiende a diferentes tipos de necesidades (médicas, psicosociales, educativas y vocacionales) cuando pasa de la atención sanitaria infantil a la de adultos. Esta nueva realidad sanitaria requiere del desarrollo de programas de transición para que la persona vaya adquiriendo gradualmente los conocimientos, habilidades y motivación necesarias para cuidar de sí misma, donde la familia y el equipo sanitario ejercerán un rol de acompañamiento^7^.

A partir de la publicación por parte de la Academia Americana de Pediatría^8^ de una serie de directrices sobre cómo guiar el proceso de transición en jóvenes con enfermedades crónicas, se produjo un aumento significativo de investigaciones centradas en la medición de la preparación para la transición. A pesar de los múltiples cuestionarios y listas de verificación empleados, las propiedades psicométricas de estas herramientas son limitadas: se centran en valorar, fundamentalmente, conocimientos y/o conductas observables^9^ sin tener en cuenta el estado emocional, elemento esencial para el mantenimiento de la salud y, al ser diseñadas en lengua inglesa, requieren de un proceso de equivalencia conceptual con el cuestionario original para garantizar su fiabilidad y validez al aplicarlos en la población española. Una transición pobremente planificada o una transferencia directa a la unidad de adultos sin mediar los pasos planificados de forma escalonada recomendados por las guías^2^ se relaciona con un deterioro en la calidad de vida y un aumento de la morbimortalidad^10^.

Las personas jóvenes con FQ experimentan los mismos cambios físicos y psicológicos que sus pares sanos, independientemente de la gravedad de su enfermedad. A esta situación hay que sumarle las alteraciones derivadas de su enfermedad: exacerbaciones respiratorias frecuentes, periodos de hospitalización, alteración de su imagen corporal relacionada con un retraso en el crecimiento, incertidumbre con respecto a su futuro en relación al deterioro de su salud, restricciones laborales, disminución de la fertilidad y de la esperanza de vida^11^. Además, el cumplimiento de las medidas terapéuticas recomendadas (tratamientos farmacológicos, fisioterapia respiratoria, citas médicas) requiere una dedicación significativa de tiempo que puede interferir en sus rutinas^12^, afectando negativamente a su estado de ánimo. Existe una relación positiva entre el estado de ánimo y la adherencia al tratamiento^13^, y una baja adherencia terapéutica se considera, a su vez, un factor de riesgo para la calidad de vida^14^.

Por todo ello, planteamos como objetivo de este estudio analizar los conocimientos, habilidades y estado emocional de los pacientes con fibrosis quística (FQ) en un momento de su seguimiento, comparándolos con el recuerdo que tenían de la transición (paso planificado desde la unidad infantil a la de adultos) o transferencia (traspaso directo a una unidad de adultos sin los pasos recomendados por las guías) a una unidad especializada de adultos.

## MATERIAL Y MÉTODOS

Estudio transversal realizado entre septiembre de 2018 y octubre de 2021 con pacientes que acudían a consultas de seguimiento en la Unidad de Fibrosis Quística de adultos del Hospital Universitario la Paz (Madrid, España).

Los criterios de inclusión fueron: pacientes con FQ^15^, mayores de edad (≥18 años) y con FQ estable (al menos un mes sin clínica de exacerbación respiratoria o complicación). Se excluyeron las personas con dificultades para cumplimentar correctamente el cuestionario y aquellas que no hubiesen firmado el consentimiento informado.

El reclutamiento de participantes fue prospectivo y secuencial según el orden de llegada a las revisiones en la consulta. Las personas que cumplían con los criterios de selección recibieron información verbal y escrita sobre su libre participación, la confidencialidad y anonimato de los datos y su uso para fines científicos.

Se obtuvo el consentimiento firmado de cada participante antes de que un miembro del equipo investigador sin relación directa con el cuidado de los pacientes recogiera los datos sociodemográficos y pasara un cuestionario autoadministrado sobre capacidad de autocuidado. La investigación se ajustó a los principios establecidos en la Declaración de Helsinki^16^ y fue aprobado por el Comité de Ética de Investigación Clínica del Hospital Universitario La Paz (código HULP: PI-330).

Para recoger los datos en la visita, el equipo investigador elaboró un documento que constaba de dos apartados:

- Datos sociodemográficos de la persona: edad (años) en el momento de hacer el estudio, edad de diagnóstico de la FQ, edad de transición a los servicios sanitarios de adultos con FQ, sexo (hombre, mujer), estado civil (soltera, casada, pareja de hecho, separada/divorciada, viuda), nivel de estudios (primarios, secundarios, formación profesional, universitarios) y ocupación (estudiante, trabajadora, estudiante y trabajadora, desempleada, pensionista). Se les preguntó si tenían reconocido algún grado de discapacidad (sí, no), si consumían sustancias tóxicas como tabaco (nunca, exfumador, ocasional, a diario), alcohol (nunca, <1 vez al mes, mensualmente, semanalmente, a diario) o cualquier otro tipo de drogas (sí, no), si realizaban actividad física (nunca, <1 vez al mes, mensualmente, semanalmente, a diario) y si realizaban fisioterapia respiratoria (nunca, <1 vez al mes, mensualmente, semanalmente, a diario).
- Encuesta sobre cómo percibían su capacidad de autocuidado en ese momento y cómo la recordaban en la transición (paso planificado y progresivo desde la unidad pediátrica de FQ a una de adultos, generalmente dentro del mismo hospital) o en la transferencia (traspaso directo desde otras unidades de FQ u otras consultas de adultos a una unidad de adultos sin mediar los pasos escalonados recomendados por las guías^2^).

La encuesta fue diseñada a partir de preguntas previamente incluidas en cuestionarios específicos que evalúan la capacidad de autocuidado^17^^-^^22^, y constaba de 22 cuestiones encuadradas en tres dimensiones. Las cuestiones correspondientes a las dimensiones *Conocimientos sobre la FQ y los cuidados necesarios para mantener la salud* (n=6) y *Habilidades comunicativas y de manejo del tratamiento* (n=11) se valoraron según una escala tipo Likert de cinco puntos (desde 0 = menor grado de conocimientos y destreza hasta 4 = mayor grado), obteniéndose la puntuación total. La dimensión *Estado emocional* (n=5) se valoró de acuerdo al dominio del mismo nombre del cuestionario revisado de calidad de vida para FQ^22^ (rango de 0 a 100, a mayor puntuación, mejor estado emocional). Cada cuestión se valoró mediante una escala tipo Likert de cuatro puntos (1 = siempre, 2 = a menudo, 3 = a veces, 4 = nunca) y la puntuación total se calculó sumando las puntuaciones de las cinco cuestiones, restando cinco y dividiendo entre 15 (se multiplicó por 100 para obtener el porcentaje)^22^.

La factibilidad de este cuestionario se determinó a través de entrevistas individuales en pacientes con FQ estable, analizando si el vocabulario era adecuado y las preguntas culturalmente aplicables al contexto español; al menos el 85% de participantes debía considerar cada cuestión comprensible y pertinente. La consistencia interna se estudió con el coeficiente *alfa de Cronbach*, (α) globalmentre y en cada una de las tres dimensiones, considerándose fiable si era ≥0,70^23^ (Anexo I).

Se dividieron los pacientes en dos grupos según cómo hubiera sido su proceso de llegada a la unidad de FQ de adultos:

- grupo 1 (transición): pacientes con varias visitas programadas, según las guías del hospital, para su correcta transición. Un especialista en Neumología adscrito a la unidad de adultos acudió a las últimas visitas realizadas en Pediatría antes de pasar a la unidad de adultos y, posteriormente, un especialista en Pediatría acompañó al participante en la primera visita en la unidad de adultos;
- grupo 2 (transferencia): pacientes derivados directamente a la unidad de adultos, sin seguir los pasos recomendados en los procesos de transición de la atención pediátrica a la de adultos en FQ^2^.

### Análisis estadístico

Los datos cualitativos se describieron mediante frecuencias absolutas y porcentajes, y los ordinales (puntuaciones) mediante la mediana (Me) y el rango intercuartílico (RIC). La normalidad de las variables continuas se estudió mediante la prueba de Kolmogorov-Smirnov, y estas se expresaron mediante la media y la desviación estándar (DE). Las comparaciones de variables continuas entre grupos independientes se realizaron mediante las pruebas t-student (prueba paramétrica) y U de Mann-Whitney (no paramétrica), y las variables categóricas mediante *Chi-cuadrado* (χ^2^) o prueba exacta de Fisher, según la frecuencia de valores observados. La evolución de las puntuaciones de las escalas ordinales del cuestionario sobre capacidad de autocuidado de cada grupo entre la transición/transferencia y el momento del estudio se realizó mediante la prueba de Wilcoxon para muestras pareadas. Todos los contrastes se consideraron bilaterales y con un nivel de confianza del 95%. El software estadístico que se empleo fue *SAS Enterprise Guide 8.2. (Cary NC, SAS Institute Inc., USA).*

## RESULTADOS

**Figura 1:**
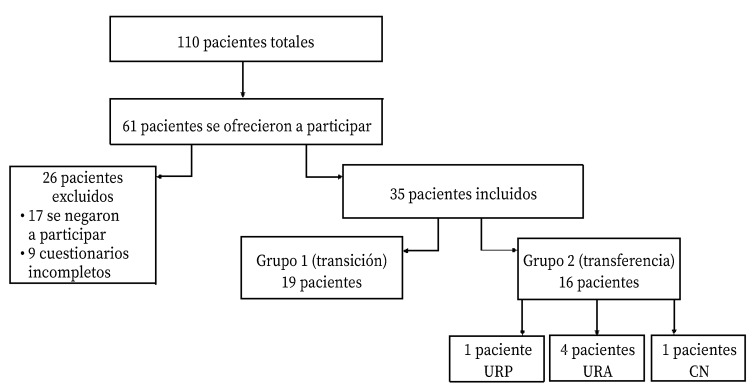
Diagrama de flujo de los participantes del estudio.

URP: unidad de referencia pediátrica; URA: unidad de referencia de adultos; CN: consultas de Neumología.

Durante la realización del estudio acudieron a una consulta 110 pacientes. De estos, aceptaron participar 61; se excluyeron 26 (42,62%), nueve por cumplimentar parcialmente los cuestionarios (34,61%) y 17 por no firmar el consentimiento informado (65,38%). La muestra de estudio incluyó 35 participantes (Fig. 1).

El 65,85% de las personas participantes eran varones, con media de edad 31,91 años (DE = 10,11). La media de edad en el momento de la transición/transferencia al servicio de adultos fue 21,69 años (DE = 7,23), habiendo transcurrido 10,23 años (DE = 7,35) hasta la visita en la que se entregó el cuestionario. El 54,28% poseían formación universitaria, menos de una cuarta parte se encontraban en situación de desempleo (5,71%) o eran pensionistas (17,14%) y un 54,29% tenía reconocido algún grado de discapacidad. Respecto a los hábitos tóxicos, la mayoría nunca habían fumado (88,57%), el 68,56% refirieron consumir alcohol nunca o menos de una vez al mes, y todas negaron consumo previo de drogas. Más de la mitad realizaba actividad física semanalmente (57,14%) y fisioterapia respiratoria diariamente (54,28%) (Tabla 1).

En el grupo 1 (transición) se incluyeron 19 pacientes (54,28%) y el grupo 2 estuvo formado por 16 personas adultas (45,71%) que fueron transferidas desde otras consultas de neumología (n=11, 31,42%), desde otras unidades de referencia de adultos de FQ (n=4, 11,42%) y desde la unidad pediátrica de FQ de otro centro (n=1, 2,85%).

Ambos grupos fueron comparables respecto a todas las variables (Tabla 1), excepto la edad de diagnóstico de la enfermedad, que fue significativamente mayor en el grupo 2 que en el grupo de transición (2,68 años; DE = 3,83 vs 15,94 años; DE = 16,45; U de Mann-Whitney p = 0,031).

**Tabla 1:** Características sociodemográficas de la muestra de estudio, global y por grupo

Características sociodemográficas	Global	Grupo 1. Transición	Grupo 2. Transferencia
	n=33	n=19 (54,28%)	n=16 (45,71%)
Edad (años), *media±DE*			
Momento del estudio	31,91 ± 10,11	30,11 ±7,39	34,06 ±12,54
Diagnostico FQ	8,74 ± 13,14	2,68 ± 3,83	15,94 ±16,45
Transición	21,69 ± 7,23	18,37 ± 1,60	22,00 ±6,22
Tiempo de seguimiento en la UAFQ (años), *media±DE*			
	10,23 ±7,35	11,74 ±7,63	8,44 ±6,80
Sexo (masculino), %	65,85	57,91	68,83
Estado civil, %			
Soltero	65,71	63,22	68,88
Casado	28,57	26,32	31,31
Pareja de hecho	5,71	10,57	0
Separado/divorciado	0	0	0
Viudo	0	0	0
Nivel de estudios, %			
Primarios	8,57	15,87	0
Secundarios	31,42	31,63	31,32
Formación profesional	5,71	0	12,58
Universitarios	54,28	52,63	56,33
Ocupación actual, %			
Estudia	14,28	10,54	18,87
Trabaja	45,71	36,86	56,34
Estudia y trabaja	17,14	21,17	12,52
Desempleo	,71	10,52	0
Pensionista	17,14	21,15	12,54
Grado de discapacidad, %	54,29	47,43	62,57
Consumo de tabaco, %			
Nunca	88,57	100	83,03
Exfumador	11,43	0	25,07
Fumador ocasional	0	0	0
Fumador a diario	0	0	0
Consumo de alcohol, %			
Nunca	31,42	26,31	37,52
Menos 1 vez al mes	37,14	47,47	25,03
Mensualmente	20,00	15,86	25,03
Semanalmente	11,42	10,58	12,52
A diario	0	0	0
Consumo de otras drogas, %	0	0	0
Realización de actividad física, %			
Nunca	8,57	10,52	6,35
Menos 1 vez al mes	14,28	10,52	18,82
Mensualmente	2,85	5,31	0
Semanalmente	57,14	63,25	50,02
A diario	17,14	10,52	25,01
Realización de fisioterapia respiratoria, %			
Nunca	11,42	10,57	12,54
Menos 1 vez al mes	5,71	5,33	6,31
Mensualmente	,57	0	18,85
Semanalmente	20	21,14	18,85
A diario	54,28	63,26	43,85

Grupo 1: pacientes que procedían de Neumología pediátrica del mismo centro sanitario (transición según los pasos recomendados por las guías^2^); Grupo 2: pacientes derivados desde otras unidades de FQ o desde otras consultas (transferencia); X ± DE: media ± desviación estándar; %: porcentaje; UAFQ: unidad de adultos de fibrosis quística; negrita: variables no comparables entre grupos según U de Mann-Whitney.

Ambos grupos entendieron bien la encuesta sobre capacidad de autocuidado (Anexo I). De las 35 personas que cumplimentaron la encuesta, 34 (97,14%) no expresaron dificultades en su comprensión cognitiva, 31 (88,57%) consideraron pertinentes las cuestiones incluidas y 32 (91,42 %) pudieron realizarla sin ayuda. Solo tres presentaron dificultad en la lectura y precisaron apoyo por parte del equipo investigador.

La consistencia interna del cuestionario fue buena (α=0,86). También se obtuvieron buenos valores de consistencia interna en las dimensiones de conocimientos (α=0,94), habilidades (α=0,85) y estado emocional (α=0,82).

En el momento de la transición/transferencia a la Unidad de FQ de adultos, ambos grupos recordaron valores similares respecto a las cuestiones de conocimientos excepto sobre su medicación, que era mayor en el grupo 1/transición. En relación con el grado de habilidades, el grupo 2/transferencia gestionaba con más facilidad sus citas y participaba mucho más activamente en la toma de decisiones aunque, respecto al estado emocional, se sentían más tristes (Tabla 2).

**Tabla 2:** Análisis comparativo de la capacidad de autocuidado (conocimientos, habilidades y estado emocional) entre los pacientes del grupo 1 y 2, en el momento de la transición/transferencia, durante el periodo de tiempo transcurrido hasta la visita del estudio y en el momento de estudio

	Transición/Transferencia			p U-MW	Momento del estudio			p U-MW	p
	Global (n=35)	G 1 (n=19)	G 2 (n=16)		Global (n=35)	G 1 (n=19)	G 2 (n=16)		Wilcoxon
1. Conocimientos sobre FQ	2 [1-3]	2 [1-3]	2 [1-3]	0,48	4 [3-4]	4 [3-4]	4 [3-4]	0,67	0,62
2. Conocimientos sobre impacto de la FQ en los órganos	2 [1-3]	2 [1-3]	2 [1-3]	0,76	4 [3-4]	4 [3-4]	4 [3,4]	0,35	0,98
3. Conocimientos sobre la medicación	2 [1-3]	3 [2-4]	2 [0-3]	0,03	4 [3-4]	4 [3-4]	3 [3-4]	0,131	0,10
4. Conocimientos sobre otras medidas	2 [1-3]	2 [1-3]	2 [1-3]	0,84	4 [3-4]	4 [3-4]	3 [3-4]	0,46	0,13
5. Conocimientos sobre signos de alerta	2 [1-3]	2 [1-3]	2 [0-2]	0,20	4 [3-4]	4 [3-4]	4 [3-4]	0,38	0,46
6. Conocimientos sobre qué hacer si necesita ayuda	2 [1-3]	3 [1-4]	2 [0-3]	0,27	4 [3-4]	4 [3-4]	3 [3-4]	0,85	0,28
7. Manejar la medicación	3 [2-4]	3 [2-4]	4 [2-4]	0,19	4 [4-4]	4 [4-4]	4 [3-4]	0,50	0,13
8. Llevar a cabo otras medidas de cuidado	3 [2-4]	2 [2-3]	3 [2-4]	0,38	4 [3-4]	4 [4-4]	4 [3-4]	0,11	0,13
9. Gestionar citas sanitarias	3 [1-4]	2 [1-3]	4 [2-4]	0,01	4 [3-4]	4 [3-4]	4 [3-4]	0,78	0,00
10. Participar en la toma de decisiones	3 [2-4]	3 [2-3]	4 [2-4]	0,04	4 [3-4]	4 [4-4]	4 [3-4]	0,10	0,01
11. Formular preguntas al equipo	3 [2-4]	3 [2-3]	4 [2-4]	0,06	4 [4-4]	4 [4-4]	4 [3-4]	0,85	0.03
12. Comunicar al equipo cambios de salud	4 [2-4]	3 [2-4]	4 [3-4]	0,06	4 [4-4]	4 [4-4]	4 [3-4]	0,50	0,03
13. Expresar miedos al equipo	3 [2-4]	2 [1-3]	3 [2-4]	0,06	4 [3-4]	4 [3-4]	4 [3-4]	0,84	0,03
14. Confiar en el equipo	3 [3-4]	3 [2-4]	3 [3-4]	0,60	4 [3-4]	4 [3-4]	4 [4-4]	0,43	0,44
15. El equipo me informa de forma comprensible	3 [3-4]	3 [3-4]	3 [3-4]	0,83	4 [3-4]	4 [3-4]	4 [3-4]	0,73	0,76
16. El equipo me responde de forma comprensible	4 [3-4]	4 [3-4]	4 [4-4]	0,14	4 [3-4]	4 [3-4]	4 [3-4]	0,93	0,25
17. El equipo es amable	4 [3-4]	4 [3-4]	4 [3-4]	0,94	4 [3-4]	4 [3-4]	4 [3-4]	0,70	0,92
18. Me he sentido preocupado	2 [2-3]	3 [2-3]	2 [2-3]	0,40	2 [1-3]	3 [2-3]	2 [2-2]	0,75	0,37
19. Me he sentido inútil	4 [3-4]	4 [3-4]	4 [2-4]	0,65	3 [3-4]	4 [4-4]	4 [3-4]	0,58	0,69
20. Me he sentido triste	3 [2-4]	3 [3-4]	3 [2-3]	0,03	3 [3-3]	4 [3-4]	3 [2-4]	0,07	0,25
21. Me he sentido solo	4 [3-4]	4 [3-4]	3 [3-4]	0,65	4 [2-4]	4 [3-4]	3 [2-4]	0,18	0,63
22. Me es difícil hacer planes de futuro	3 [2-4]	3 [2-3]	3 [2-3]	0,39	3 [2-4]	3 [2-3]	2 [2-3]	0,47	0,73
Puntuación total del estado emocional	66,23	72,28	57,92	0,07	72,80	80,40	63,80	0,02	0,18
	-21,44	-17,25	-25,84		-23,23	(18,92)	(25,18)		

Todos los valores se expresan como mediana ± rango intercuartílico y se comparan con U de Mann-Whitney excepto la puntuación total del estado emocional (media y desviación estándar), que se compara mediante t de Student. G 1: pacientes que procedían de Neumología pediátrica del mismo centro sanitario (transición según los pasos recomendados por las guías^2^); G 2: pacientes derivados desde otras unidades de fibrosis quística o desde otras consultas (transferencia); resultados en negrita: diferencias significativas entre grupos en el momento de su transición/transferencia, en el momento del estudio y en la evolución.

En el momento de la realización del estudio, ambos grupos declararon el mismo grado de conocimientos y de habilidades. Sin embargo, la puntuación del estado emocional fue significativamente superior en el grupo 1 (Tabla 2).

Al analizar el cambio producido en cada grupo desde su transición/transferencia hasta el momento del estudio, se observó que, con el paso del tiempo, el grupo 1 aprendió a gestionar sus citas de forma autónoma, a comunicarse de forma constructiva con el equipo sanitario y a participar en la toma de decisiones en relación con su salud (Tabla 2). Estos resultados no variaron significativamente al analizar los datos desagregados por sexo (Material suplementario).

## DISCUSIÓN

La transición de la atención pediátrica a la de adultos, en líneas generales, se produce en un periodo de numerosos cambios físicos y psicológicos asociados a la juventud, por lo que a esta situación vital, compleja, se le suma el cambio en la atención sanitaria. Los pacientes transferidos directamente desde otras unidades de FQ o consultas de adultos inician su seguimiento sin realizar los pasos escalonados recomendados por las guías^2^. Ambas situaciones (transición/transferencia) requieren conocimientos y habilidades suficientes y un estado emocional estable para promover la continuidad de cuidados en la unidad de adultos de FQ.

En nuestro estudio hemos observado que, en el momento de la transición, el grupo 1 (transición escalonada) presentaron un mayor nivel de conocimiento sobre las medicaciones prescritas, probablemente debido a que, en este grupo, la enfermedad se diagnosticó en la infancia, lo que ha permitido conocer muchos aspectos de la enfermedad y entre ellos la medicación recomendada^24^. En el seguimiento, ambos grupos mejoraron sus puntuaciones en los conocimientos y habilidades, en gran parte, gracias a la labor informativa-educativa del equipo dedicado a su cuidado. Otros estudios que valoraron el grado de conocimientos sobre patologías crónicas en el proceso de la transición de la atención pediátrica a la de adultos^17^, y en concreto en FQ^20^, observaron una relación positiva entre la edad y los conocimientos sobre el manejo de su enfermedad, aunque el rango etario oscilaba entre diez y 19 años. No hemos encontrado estudios que exploren los aspectos cognitivos del autocuidado en personas adultas y que nos permitan comparar aquellas que se trataron en la infancia y vivieron una transición escalonada con visitas específicas a la unidad de adultos con aquellas diagnosticadas en la edad adulta o que, como en el caso de nuestro estudio, han retomado en la adultez el seguimiento clínico tras una interrupción del tratamiento.

El grupo 2 (pacientes que vivieron una transferencia directa) presentó mayores habilidades en la gestión de citas sanitarias y toma de decisiones, resultados muy similares a los de Lapp y col^25^ sobre la percepción de jóvenes con FQ sobre sus habilidades en el momento de la transición y que observaron que la edad se relacionaba con la destreza en el manejo de citas y con las habilidades comunicativas con los profesionales. En cambio, el grupo 1 refirió que la tarea de gestionar citas era de las asumidas más tardíamente, al realizarla sus progenitores por costumbre y a que muchos se sentían cómodos en dicho rol. Sin embargo, al analizar la evolución de los grupos entre el momento de la transición/transferencia y el momento del estudio, se observó que el grupo 1 mejoró significativamente en la gestión autónoma de citas, comunicación y participación activa en la toma de decisiones relacionadas con su salud, equiparándose al grupo 2. Diferentes autores^10^ reconocen la relevancia de fomentar la comunicación y desarrollar una actitud proactiva en la toma de decisiones para lograr una continuidad de cuidados exitosa.

Además de los conocimientos y habilidades para cuidarse, la motivación también es un elemento influyente en la salud de las personas con enfermedades crónicas, y aquí juega un papel crucial el estado emocional, puesto que un ánimo bajo constituye una barrera para el autocuidado de personas con problemas de salud crónicos^26^. Concretamente, el estudio epidemiológico internacional sobre prevalencia de depresión y ansiedad en pacientes con FQ^13^ demostró una alta tasa de síntomas depresivos en la cohorte española respecto de la población general^27^. Nuestros resultados fueron consistentes con los de otros estudios publicados^13^^,^^14^ que asociaron mayor edad con estado emocional más bajo. La adquisición progresiva de habilidades de comunicación (expresar verbalmente cómo se encuentra, expresar sus miedos, formular preguntas al equipo y ser partícipe en la toma de decisiones) y de gestión de citas pudo contribuir positivamente al mejor estado emocional del grupo 1.

Aunque en el grupo 2 predominaron las personas diagnosticados en la etapa adulta, la edad media global de transición/transferencia a la unidad de FQ de adultos fue 21,7 años, muy similar a la observada en el registro español de FQ (20,7)^28^ y en el registro europeo de FQ (20,9)^3^.

La frecuencia de personas con FQ con formación universitaria fue nueve puntos superior a la de la población general española de entre 25 y 44 años^29^ (45,5%), lo que corrobora que presentar una enfermedad crónica no es una limitación para iniciar estudios superiores. En la edad adulta, tan importante es la formación como el desempeño profesional; la tasa de desempleo de la muestra fue (5,71%) inferior a las obtenidas por Olveira y col^14^ en personas con FQ realizada por (29,9) o por el Instituto Nacional de Estadística en población general española de 20 a 44 años^30^ (11,4%). La discrepancia podría deberse a que el 54,3% de los sujetos que participaron tenían reconocido un grado de discapacidad de al menos un 33%, que posibilita el acceso a un empleo protegido (modalidad laboral adaptada a las capacidades de las personas con minusvalía).

Coincidiendo con otras publicaciones previas^21^^,^^31^, no encontraron diferencias significativas por sexo respecto a la capacidad de autocuidado, ni en la transición/transferencia ni en el momento del estudio.

La principal limitación metodológica del estudio es la posible existencia de que, en la misma visita durante el seguimiento ambulatorio, la persona cumplimentó la misma encuesta dos veces: una analizó su capacidad para el autocuidado (conocimientos de la enfermedad, habilidades y estado emocional) en el momento actual y la otra recogió sus recuerdos sobre la capacidad de autocuidado en su transición/transferencia, lo que podría suponer un sesgo de memoria. Sin embargo, pese a esta limitación, obtuvimos diferencias significativas entre grupos y en la evolución de cada grupo, lo que nos ha permitido conocer cómo mejorar los pasos recomendados en la transición^2^ y cómo deberíamos llevar a cabo la transferencia de las personas que acuden a una unidad de FQ de adultos. Otra limitación es que se trata de un estudio unicéntrico, lo que podría comprometer su validez externa debido a las características de la muestra. A pesar de que el número de participantes fue pequeño (la FQ es una enfermedad rara con una baja prevalencia registrada en España^28^), los resultados obtenidos han sido similares a los publicados^(22, 25)^. Además, la presentación y acompañamiento en la cumplimentación de la encuesta por parte de un profesional ajeno al equipo de FQ de adultos podría haber contribuido a una menor participación, si bien ha aportado objetividad en el momento de la recogida de datos. El tiempo disponible por parte de la persona con FQ no fue, a veces, suficiente para contestar el total de las preguntas del cuestionario, alrededor de 30 minutos, por lo que esos pacientes se excluyeron. Otra limitación fue la ausencia de una herramienta validada y adaptada a población española que evaluase la preparación para la transición a la unidad de adultos de FQ. Por ello, se diseñó una encuesta nueva que está en vías de validación y se pasó la primera parte en enfermos estables (al menos, un mes sin clínica de exacerbación), evaluando su factibilidad y consistencia interna.

En conclusión, las personas con FQ derivadas desde la unidad pediátrica especializada del mismo centro sanitario refirieron mayor conocimiento sobre la medicación, menores habilidades de gestión de citas y menor participación en la toma de decisiones en el momento de la transición que las personas derivadas a la unidad de FQ de adultos desde otras unidades de FQ o desde otras consultas de adultos en el momento de la transferencia. En el momento actual, las personas transferidas poseían peor estado emocional. Dado que el estado emocional es un elemento clave para la adherencia al tratamiento, su valoración es relevante.

Dado que la bibliografía se centra, principalmente, en la preparación de personas con FQ para la transición de la atención pediátrica a la de adultos, nuestro estudio aporta conocimiento sobre la capacidad de autocuidado de pacientes directamente transferidos a una unidad especializada de adultos. Además, este estudio supone una primera aproximación a la evaluación del grado de conocimientos, habilidades y estado emocional de personas adultas que están en seguimiento por una unidad de referencia en FQ.

## Data Availability

Los datos están disponibles bajo petición razonada a la autora de correspondencia.

## ANEXO I Encuesta sobre capacidad de autocuidado en adultos con fibrosis quística

Señale el **grado de conocimientos** que tenía en el momento de la transición del servicio de atención pediátrica al equipo de adultos o cuando llegó a la unidad de fibrosis quística (FQ) de adultos y, en el momento actual, de acuerdo con una escala de 0 a 4:

0 = NINGÚN CONOCIMIENTO

1 = CONOCIMIENTO ESCASO

2 = CONOCIMIENTO MODERADO

3 = CONOCIMIENTO SUSTANCIAL

4 = CONOCIMIENTO EXTENSO

NC = NO CONTESTA

**Table:**

	Transición/Llegada						Actualidad					
	Unidad FQ Adultos												
	0	1	2	3	4	NC	0	1	2	3	4	NC
1. Conocimientos sobre qué consiste la FQ y su pronóstico												
2. Conocimientos sobre el efecto que tiene la FQ en el sistema respiratorio, aparato digestivo, órganos sexuales												
3. Conocimientos sobre la medicación: qué son, para qué sirven, cuando tomarlos, efectos adversos												
4. Conocimientos sobre otras medidas terapéuticas como la nutrición, fisioterapia respiratoria o el ejercicio físico												
5. Conocimientos sobre signos de alerta que pueden indicar un empeoramiento de su salud												
6. Conocimientos sobre qué hacer si necesita ayuda												

Señale el **grado de gestión de sus cuidados** que tenía en el momento de la transición del servicio de atención pediátrica al equipo de adultos o cuando llegó a la unidad FQ de adultos y, en el momento actual, de acuerdo con una escala de 0 a 4:

0 = NADA CAPAZ

1 = ALGO CAPAZ

2 = MODERADAMENTE CAPAZ

3 = MUY CAPAZ

4 = COMPLETAMENTE CAPAZ

NC = NO CONTESTA

**Table:**

	Transición/Llegada Unidad FQ Adultos						Actualidad					
	0	1	2	3	4	NC	0	1	2	3	4	NC
7. Manejar de forma autónoma la administración de la medicación, así como posibles modificaciones de la misma												
8. Llevar a cabo las medidas de nutrición, fisioterapia respiratoria y ejercicio respiratorio de forma autónoma												
9. Gestionar de forma autónoma las citas sanitarias necesarias para el seguimiento												
10. Participar en la toma de decisiones en relación con su salud												
11. Formular preguntas al equipo sanitario de adultos												
12. Comunicar al equipo sanitario de adultos los cambios que se producen en su salud												
13. Expresar al equipo sanitario de adultos sus preocupaciones y miedos												
14. Confiar en el equipo sanitario de adultos												
15. El equipo sanitario de adultos me informaba de forma comprensible a mí y a mi familia												
16. El equipo sanitario de adultos respondía de forma comprensible a mis dudas y de mi familia												
17. El equipo sanitario de adultos era amable conmigo y mi familia												

Señale la **frecuencia de las siguientes afirmaciones**, de acuerdo con una escala de 1 a 4, durante la transición o cuando llegó a la unidad FQ de adultos y en el momento actual:

1 = SIEMPRE

2 = A MENUDO

3 = A VECES

4 = NUNCA

NC = NO CONTESTA

**Table:**

	Transición/Llegada Unidad FQ Adultos					Actualidad				
	1	2	3	4	NC	1	2	3	4	NC
18. Me he sentido preocupado/a										
19. Me he sentido inútil										
20. Me he sentido triste										
21. Me he sentido solo/a										
22. Me resulta difícil hacer planes de futuro										
